# Decreased Soluble Human Leukocyte Antigen E Levels in Patients After Allogeneic Hematopoietic Stem Cell Transplantation Are Associated With Severe Acute and Extended Chronic Graft-versus-Host Disease and Inferior Overall Survival

**DOI:** 10.3389/fimmu.2019.03027

**Published:** 2020-01-10

**Authors:** Lambros Kordelas, Esther Schwich, Monika Lindemann, Falko M. Heinemann, Ulrike Buttkereit, Peter A. Horn, Dietrich W. Beelen, Vera Rebmann

**Affiliations:** ^1^Department of Bone Marrow Transplantation, University Hospital Essen, Essen, Germany; ^2^Institute for Transfusion Medicine, University Hospital Essen, Essen, Germany

**Keywords:** allogeneic hematopoietic stem cell transplantation, graft-versus-host disease, GvHD biomarker, HLA, HLA-E, HLA-G

## Abstract

HLA-E is a member of the non-classical HLA molecules and by interaction with activating or inhibitory receptors of NK and T cells, HLA-E can lead to immune activation or suppression context-dependently. Recently, the non-classical HLA molecules gain more attention in the setting of allogeneic hematopoietic stem cell transplantation (HSCT). Most studies so far have focused on the two most frequent genotypes (HLA-E*01:01 and HLA-E*01:03) and investigated their potential association with clinical endpoints of HSCT, like graft-versus-host disease (GvHD), relapse, and overall survival (OS). However, these studies have produced inconsistent results regarding the role of HLA-E and the clinical endpoints after HSCT. We therefore here investigate the amount of soluble HLA-E (sHLA-E) in patients following HSCT and relate this to the clinical endpoints after HSCT. In univariate analysis, we observe a significant association of reduced levels of sHLA-E with severe acute GvHD, extended chronic GvHD and with inferior OS. Using receiver operating characteristic analyses specific thresholds obtained 1, 2, or 3 month(s) after HSCT were identified being indicative for severe acute GvHD, extended chronic GvHD, or inferior OS. In sub-group analyses, this effect can be confirmed in patients not treated with ATG, but is derogated in ATG-treated patients. Notably, we could not detect any association of the course of sHLA-E levels post-HSCT with the three most frequent HLA-E genotypes (HLA-E*01:03/*01:03, HLA-E*01:01/*01:01, HLA-E*01:01/*01:03). However, with regard to 5-year-OS there was an association of HLA-E*01:03 homozygosity with inferior OS. Taking ATG-treatment, recipient and donor HLA-E genotypes into consideration among other well-known risk factors, the sHLA-E status was found as an independent predictor for the development of extended cGvHD and inferior OS following HSCT irrespective of the sHLA-E thresholds. These findings shed some light on the possible impact of reduced sHLA-E levels after HSCT on GvHD and OS. Thus, sHLA-E appears to be a novel promising candidate for the prediction of clinical HSCT outcome with regards to extended cGvHD and OS.

## Introduction

Immunological processes of self-recognition and tolerance are essential for the success of allogeneic hematopoietic stem cell transplantation (HSCT) and the reconstitution of donor-derived hematopoiesis. Moreover, immunological processes significantly influence the degree of morbidity and mortality after HSCT, as they determine the incidence and severity of Graft-versus-Host-Disease (GvHD). The Human Leukocyte Antigens (HLA) system plays a central role in the innate and adaptive immune system. HLA molecules are divided into classes I and II. In both classes, classical and non-classical molecules are distinguished: The classical molecules are HLA-A, -B and -C in class I (“Ia”) and HLA-DR, -DQ and -DP in class IIa. In contrast to these, the non-classical molecules in class I (“Ib”) are HLA-E, -F and -G, MICA and MICB and in class IIb HLA-DM and -DO (1). For many years, the classical HLA-molecules have been in the focus. Recently, there is growing evidence for the importance of non-classical HLA molecules in HSCT.

HLA-E is the least polymorphic of all HLA class I molecules and acts as a ligand for the innate and adaptive immune system (2). There are 15 alleles described encoding six proteins, but only two phenotypes (HLA-E*01:01 and HLA-E*01:03) exist at high frequency (3, 4). In contrast to the ubiquitously expressed classical HLA class I proteins, the expression of the non-classical HLA-E is restricted to specific cells. In non-lymphoid organs, HLA-E protein expression is mainly restricted to endothelial cells (EC); in lymphoid organs (lymph nodes, spleen) HLA-E is strongly expressed in B and T lymphocytes, NK cells, and macrophages (5) By interaction with activating and inhibitory receptors of NK and T cells, HLA-E can lead to immune activation or suppression. HLA-E is the pre-dominant ligand for the inhibitory NK cell receptor CD94/NKG2A that prevents NK-cell-mediated lysis (6). In contrast, binding of HLA-E with the activating NK cell receptor CD94/NKG2C delivers an activation signal to NK cells. HLA-E shows a six-fold higher affinity to the inhibitory CD94/NKG2A receptor than to the activating CD94/NKG2C receptor (7).

Similar to HLA-G, HLA-E is expressed during pregnancy on trophoblasts and contributes to the tolerance against the “semi-allograft” fetus by engagement with inhibitory NK cell receptors (8–10). Moreover, there is ample evidence for the involvement of HLA-E in immune surveillance but also in immune evasion mechanisms in virally infected or malignantly transformed cells (1). In virally infected cells HLA-E is “hijacked” to up-regulate HLA-E expression to mimic normal levels and thus to escape from immune surveillance (4). HLA-G and HLA-E interaction establishes an immunosuppressive microenvironment, which facilitates escape from tumor surveillance (9). Several reports describe worse outcome in solid tumors if HLA-G and HLA-E are co-expressed [reviewed in (9)]. Recently, elevated soluble HLA-E plasma levels were associated also with worse outcome in hematological malignancies (11).

Given its context-dependent immune modulation by interacting with activating or inhibitory receptors, the role of HLA-E has also been investigated in the context of HSCT. However, the studies have described partly contradictory results regarding the influence of the three most frequent genotypes (HLA-E*01:01/01:01, HLA-E*01:01/01:03, HLA-E*01:03/01:03) on transplant-related mortality (TRM), acute, and chronic GvHD, relapse, disease free survival (DFS), and overall survival (OS) after HSCT (12–22). We have previously observed in a cohort of 32 patients that elevated soluble HLA-G levels after HSCT are associated with less severe acute and chronic GvHD (23). Here, we investigated soluble HLA-E (sHLA-E) levels in 93 patients post-HSCT and HLA-E genotypes of donor and recipient pairs. The results were related to relevant clinical outcomes of HSCT like acute and chronic GvHD, relapse, and OS.

## Materials and Methods

### Study Design and Patient Recruitment

This monocentric study was planned prospectively, approved by the Ethical Board of the University Hospital of Essen (07-3503) and carried out in accordance to the Declaration of Helsinki. All patients signed a written consent form to participate in this study. We categorized acute and chronic GvHD according to accepted standards (24–26). Ethylenediaminetetraacetate (EDTA) plasma samples were serially procured from the patients 1, 2, 3, 4, 5, 6, 9, and 12 month(s) before and after transplantation.

### Patients' and HSCT-Characteristics

Ninety-three patients, 49 female and 44 male, were enrolled in the study. Median age was 54 years (range 19–75 years). The majority [55 patients (pts.)] were diagnosed with Acute Myeloid Leukemia (AML). Other diagnoses included Myelodysplastic Syndrome (MDS, 10 pts.), Acute Lymphoblastic Leukemia (ALL, 5 pts.), Non-Hodgkin Lymphoma (NHL, 5 pts.), Myeloproliferative Neoplasms (MPN, 6 pts.) and other (12 pts.). These patients underwent HSCT between October 2008 and December 2018 at the Department of Bone Marrow Transplantation of the University Hospital Essen, Germany. Median CD34^+^ transplanted was 7.0 × 10^6^/kg body weight (BW) of the recipient (range 3.0–19.5). The patients' and HSCT characteristics are detailed in Table 1. Fifty-four of the ninety-three patients received anti-thymocyte globulin (ATG) as *in vivo* T-cell depletion. At our center, ATG was given in case of unrelated donors. Hence, out of the total 54 cases with ATG, the majority of HSCTs were with matched unrelated (MUD) and mismatched unrelated donors (MMUD). Only in three cases with related donors, ATG was applied for exceptional and individual reasons (haplo-identical donor; CD34+ positive selection and no other GvHD prophylaxis; putatively anti-proliferative effect in T-NHL).Twenty-nine patients received total body irradiation (TBI) as part of the conditioning regimen. Twenty-seven patients received grafts from related donors; the remaining 66 patients received grafts from unrelated donors. In 76 cases the HSCT was HLA-identical, 17 patients were transplanted with an HLA-mismatched graft. ATG- and non-ATG-cohorts were largely equally distributed. There was no significant difference in median age, gender, diagnoses, CD34^+^ cells/kg BW, conditioning regimes, HLA-identical vs. mismatched, gender mismatch, acute GvHD grade 0-I vs. II-IV, no/limited vs. extended chronic GvHD, relapse and OS when comparing the ATG- and the non-ATG-cohort. Only the frequency of unrelated donors was significantly higher in the ATG-cohort and the GvHD prophylaxis differed significantly in the two cohorts (Table 1). At a median follow-up of 427 days (range: 38–3,874) after HSCT, 12 patients (13%) had suffered a relapse and 62 patients (67%) were alive.

**Table 1:** Demographic and HSCT characteristics of patients.

	**All patients**	ATG-treated^*^	**Non-ATG-treated**	*p*-value^**^
Number of patients	93	54	39	
Median age [years (range)]	54 (19–75)	57 (19–75)	50 (20–69)	n.s.
Gender (female/male)	49/44	30/24	19/20	n.s.
Diagnosis at alloSCT				n.s.
AML	55	30	25	
MDS	10	8	2	
ALL	5	2	3	
NHL	5	3	2	
MPN	6	3	3	
Other	12	7	5	
CD34 × 10^6^/kg BW [median (range)]	7.0 (3.0–19.5)	6.9 (3.0–15.0)	7.1 (3.1–19.5)	n.s.
Conditioning				n.s.
TBI (8–12 Gy) & flu, Cycloph or Etopos	29	15	14	
Fludarabine and busulfan	27	20	7	
Fludarabine and treosulfane	24	16	8	
Fludarabine, thiotepa ± melphalan	9	2	7	
Other	4	2	2	
Donor related/unrelated	Dec-66	3/51	24/15	<0.0001
HLA-identical yes/no	76/17	48/6	28/11	n.s.
Female donor to male patient yes/no	12/81	4/50	8/31	n.s.
Follow-up time after allo-SCT [days (median, range)]	427 (38–3874)	419 (55–3640)	427 (38/3874)	n.s.
GvHD prophylaxis				0.0067
CSA and MTX	60	43	24	
MMF and CSA/Steroids	16	5	11	
Steroids ± CSA	11	2	9	
Other/None	6	4	2	
GvHD^***^				
Acute GvHD grade 0–I	58	34	24	n.s.
Acute GvHD grade II–IV	35	20	15	
No or limited chronic GvHD	68	27	41	n.s.
Extended chronic GvHD	17	10	7	
Relapse (yes/no)	12/81	9/45	3/36	n.s.
Survival (yes/no)	62/31	36/18	28/11	n.s.

* *All but one patient received ATG Neovii™ in a cumulative dosage of 30–60 mg/kg BW. One patient received Thymoglobulin Genzyme™ in a dosage of 6 mg/kg BW*.

** *Comparisons between patients treated with ATG and non-treated with ATG (Fisher's exact test or unpaired t-test); n.s., not significant*.

*** *GvHD not evaluated for all patients due to death/missing clinical data*.

### Definitions of Disease Stage

Disease stage was classified according to the EBMT risk score for outcome after HSCT (27). Briefly, early disease stage included acute leukemia (AL) transplanted in first complete remission (CR), MDS either untreated or in first CR, NHL, and multiple myeloma (MM) transplanted untreated or in first CR. Intermediate stage included AL in second CR, MDS in second CR or in partial remission (PR), NHL and MM in second CR, in PR or in stable disease. All other disease stages were considered as late stages.

### Quantification of sHLA-E

Determination of soluble HLA-E levels was performed as previously described (11). To capture sHLA-E the monoclonal antibody (mab) 3D12 (eBioscience, Frankfurt, Germany) was used in a final concentration of 6.7 μg/ml. Plasma samples were diluted 1:2 in PBS and tested in duplicate. Purified HLA-E*01:03 protein served as standard reagent (28).The concentration of standard reagent ranged from 0.0 to 4,000 pg/ml. For the detection of bound molecules biotinylated anti-HLA-E mab (MEM/07, Exbio, Praha, Czech Republic) was used in a final dilution of 1.25 μg/ml, followed by Streptavidin HRP (R&D, Minneapolis, USA). 3,3,5,5 tetramethylbenzidine Super Slow (Sigma, Munich, Germany) served as substrate solution. After 30 min. the enzyme reaction was stopped with 1 M H_2_SO_4_ and the optical density was measured at 450 nm (Biotek Instruments, Winooski, VT). Determination of sHLA-E plasma levels was performed by four-parameter curve fitting. The intra- and inter-assay variation of the ELISA was 5.5 and 11.9%, respectively.

### HLA-E Genotyping

Recipients and corresponding donors were typed for HLA-E with a sequence-specific primer-(SSP)-PCR, as described previously. Genomic DNA was isolated from buffy-coats of peripheral blood using QIAamp® DNA Blood Mini Kit (QIAGEN GmbH, Hilden, Germany) and adjusted to a final concentration of 50 ng/μl. HLA-E amplifications were performed in a Geneamp 9700 PCR thermal cycler (Applied Biosystems, Waltham, USA) DNA (50 ng) in a final volume of 10 μl containing 3 μl PCR Master Mix (Olerup® SSP AB, Stockholm, Sweden), 0.6 units of Taq DNA Polymerase (QIAGEN GmBH), 15 pmol of detection primers, and 15 pmol of each positive control primer. The initial denaturation of the sequence-specific products was performed for 2 min at 94°C, followed by a two stage PCR program: 10 cycles of 10 s at 94°C and 20 s at 65°C and 20 relaxed cycles of 10 s at 94°C, of 1 min at 61°C, and of 30 s at 72°C. HLA-E alleles were identified at the resolution level of the second field. Human growth hormone was used as a positive control for PCR amplification (29, 30). The HLA-E genotype distributions of recipients and donors fit to the expectations of Hardy-Weinberg equilibrium.

### Statistics

Statistical analyses and presentation were performed by using SPSS 23.0 (SPSS Inc., Chicago, IL, USA) or GraphPad Prism V8.1.2 software (GraphPad Software, San Diego, CA, USA). Data are presented as mean ± SEM (standard error of mean). After testing for Gaussian distribution, continuous variables were compared by *T*-test and non-parametric Mann-Whitney or two-way analysis of variance. For categorical data, 2-sided Fisher's exact test was used. Using BIAS 11.08 software program (https://www.bias-online.de/) receiver operating characteristic (ROC) analysis was performed to define the optimal threshold value for sHLA-E regarding sensitivity and specificity for the prediction of aGvHD, cGvHD, and OS. In univariate analysis of time-to-event, the probabilities of the patients' OS were estimated by the Kaplan-Meier method and compared using log-rank between groups of interest. Stepwise multivariate Cox regression according to proportional hazards assumption or binomial logistic regression was used to identify prognostic factors for OS and cGvHD, respectively. Covariates were included into the multivariate analyses based on conceptual evaluation of literature or being associated with a *p* < 0.05 to certain clinical parameters in univariate analysis. Statistical significance was defined as *p* ≤ 0.05.

## Results

### Reduced sHLA-E Levels Are Associated With Severe Acute and Extended Chronic GvHD and Inferior OS Following HSCT

**Figure 1:**
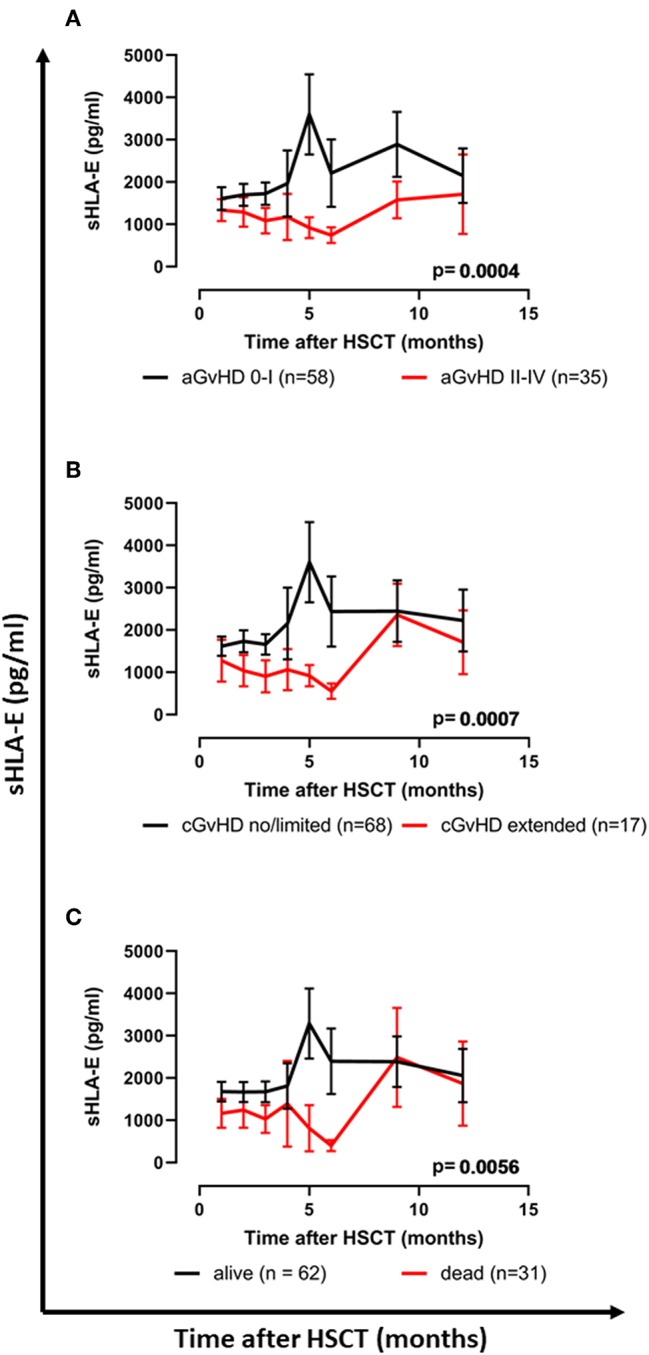
Association of reduced sHLA-E levels with severe acute, extended chronic GvHD, and inferior OS following HSCT. **(A)** The courses of sHLA-E levels in patients with **(A)** aGvHD grade II–IV (red line) vs. aGvHD grade 0–I (black line), **(B)** extended cGvHD (red line) vs. no/limited cGvHD (black line), or **(C)** patients having died (red line) vs. patients being alive (black line) during the follow-up time. Data are presented as mean ± SEM. Manifestation of aGvHD or cGvHD could not be evaluated for all patients due to death or missing clinical data.

Pre-HSCT it appeared that sHLA-E levels were independent of the patients' gender, HLA-E genotype, and disease of the patients. No significant difference of the sHLA-E levels were observed pre-HSCT and the first month post-HSCT (Supplementary Figures 1A–D). Overall, the course of sHLA-E plasma levels did not substantially vary over the observation period of 12 months post-HSCT (Supplementary Figure 2). However, patients (*n* = 35) experiencing moderate to severe aGvHD grade II–IV after HSCT displayed significantly (*p* = 0.0004) reduced sHLA-E levels (mean ± SEM) compared to patients (*n* = 58) without or with only mild acute GvHD (aGvHD 0-I, Figure 1A). Similarly, sHLA-E levels were significantly (*p* = 0.0007) diminished in patients (*n* = 17) with extended chronic GvHD compared to patients (*n* = 68) without or with limited cGvHD (Figure 1B). Furthermore, lower sHLA-E levels were significantly associated with the mortality post-HSCT (*p* = 0.0056, Figure 1C). The course of sHLA-E levels post-HSCT was not associated with relapse post-HSCT (data not shown).

### Threshold Values of sHLA-E Indicative for Severe Acute and Extended Chronic GvHD Can Be Identified

To establish sHLA-E levels as an early biomarker for the prediction of clinical outcome, the optimal threshold values in terms of sensitivity and specificity were defined for sHLA-E levels obtained 1, 2, and 3 month(s) post-HSCT by ROC analysis (Supplementary Figure 3; Supplementary Table 1). The sHLA-E cut-off levels of month 1 and 2 were not found to be associated with aGvHD, whereas sHLA-E cut-off of month 3 (cut-off: 652 pg/ml, sensitivity: 63.3%, specificity: 68.5%, AUC: 0.655; *p* = 0.019) related to severe aGvHD grade II-IV post-HSCT. Using this cut-off value, an Odd-Ratio (OR) of 3.8 with 95% confidence interval (95% CI) of 1.4–9.2 was obtained (Table 2). Considering cGvHD, sHLA-E levels below 450 pg/mL in the first month, below 523 pg/mL in the second month, or below 652 pg/mL in the third month post-HSCT were significantly indicative for patients developing extended cGvHD with ORs (Table 2) of 3.7 (95% CI: 1.2–10.1, *p* = 0.034), 4.2 (95% CI: 1.3–14.0, *p* = 0.018), and 6.0 (95% CI: 1.5–21.2, *p* = 0.007), respectively.

**Table 2:** Association of sHLA-E cut-off levels with aGvHD, cGvHD, and 5-year overall survival (OS).

**HSCT endpoint**	**sHLA-E cut-off**	***p***	**OR**	**95% CI**
aGvHD	1,595	>0.999	0.889	0.335–2.315
	608	0.113	2.167	0.859–5.031
	652	0.006	3.759	1.409–9.180
cGvHD	450	0.034	3.656	1.173–10.05
	523	0.018	4.211	1.342–14.03
	652	0.007	6.013	1.516–21.23
OS	450	0.013	3.643	1.442–9.704
	523	0.012	3.656	1.388–9.156
	1,244	0.301	1.907	0.714–5.168

*Cut-offs were given in pg/ml, p-values were calculated by Fisher's exact test. OR, odds ratio; CI, confidence interval*.

### Association of Low sHLA-E Plasma Levels at Month 1 and 2 Post-HSCT With Reduced Probabilities of OS

**Figure 2:**
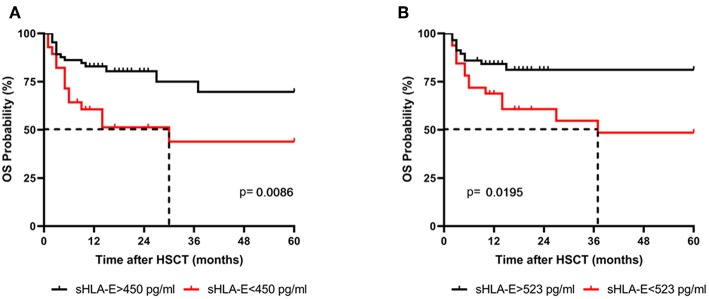
Association of low sHLA-E levels with reduced 5-year OS probabilities. Patients were divided in two groups according to cut-off levels obtained **(A)** 1 and **(B)** 2 month(s) post-HSCT. Patients with sHLA-E levels below the thresholds (red line) had a significantly reduced 5-year OS by Kaplan-Meier analysis combined with log-rank test. Dashed line indicates the median survival time.

Levels of sHLA-E below 450 pg/mL in the first month were also significantly associated with mortality, as defined by ROC analysis (sensitivity: 50.0%, specificity: 78.5%, AUC: 0.639; Supplementary Figure 3; Supplementary Table 1) with an OR of 3.6 (95% CI: 1.4–9.7, *p* = 0.013, Table 2). Kaplan-Meier probabilities of 5-years OS were reduced with a median survival of 30 months for patients below this cut-off level (*n* = 28) compared with patients having sHLA-E levels >450 pg/mL (*n* = 65) with an undefined median survival time (*p* = 0.0086, log-rank Hazard Ratio (HR) 2.58, 95% CI of ratio 1.12 to 5.89, Figure 2A). Similarly, using a sHLA-E cut-off of 523 pg/mL (sensitivity: 58.3%, specificity: 72.5%, AUC: 0.651; Supplementary Figure 3; Supplementary Table 1) calculated as optimal threshold level for the second month post-HSCT, the probability of 5-year OS was significantly lower for patients below this value (*n* = 32) with a median survival of 37 months than for patients above this level (*p* = 0.0195, HR: 2.6, 95% CI: 1.1–7.1, *n* = 57, Figure 2B).

### The Course of sHLA-E Levels Is Not Associated With the HLA-E Genotypes, but Donor HLA-E*01:03/*01:03 Genotype Is Associated With Reduced Probabilities of 5-Years-OS

**Figure 3:**
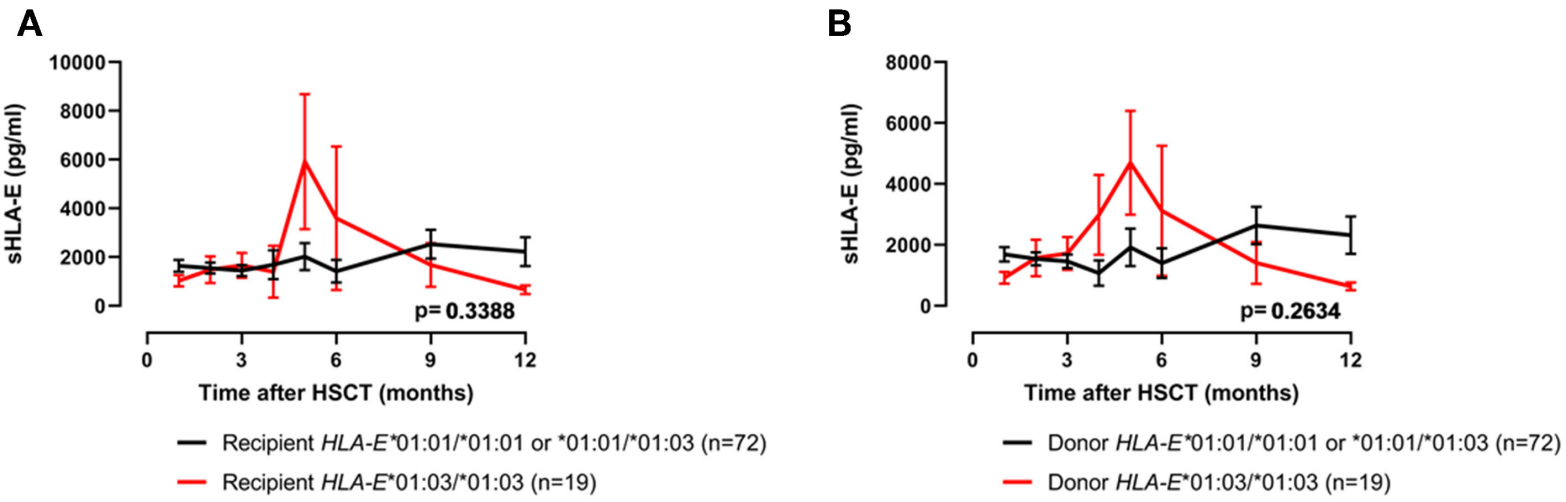
No relationship of sHLA-E post-HSCT and HLA-E genotypes. Patients' sHLA-E levels were divided into two groups according to the HLA-E*01:03/01:03 status genotype (red line) of the recipient **(A)** or of the donor **(B)**. Data are presented as mean ± SEM.

**Figure 4:**
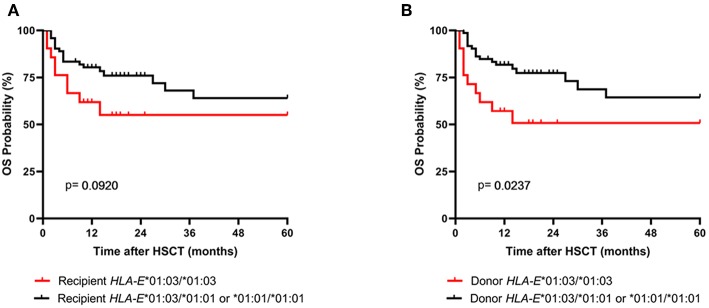
Association of the donor HLA-E*01:03/*01:03 genotype with reduced probabilities of OS. Patients were divided in two groups according **(A)** to the recipient HLA-E genotype status or **(B)** to the donor HLA-E genotype status. In Kaplan-Meier analysis combined with log-rank test the donor HLA-E*01:03/*01:03 genotype (**B**, red line) but not the one of the recipient showed a significantly reduced 5-year OS post-HSCT.

As the amount of sHLA-E plasma levels has been associated with certain HLA-E genotypes, HLA-E typing was performed for recipients and donors (10). The allele and genotype frequencies were comparable with previous reports and they did not differ between recipients and donors (Supplementary Table 2) (3, 22). The course of sHLA-E levels post-HSCT was neither associated with the recipient nor with the donor HLA-E genotypes HLA-E*01:03/*01:03 and HLA-E*01:01/*01:01 or HLA-E*01:01/*01:03 (Figures 3A,B). Moreover, neither the frequencies of HLA-E genotypes nor the frequencies of alleles of recipient and donor were significantly related to the OS post-HSCT (Supplementary Table 2). However, taken the time course into consideration the Kaplan-Meier curve analysis combined with log-rank revealed that the 5-year OS probability was significantly reduced for patients (*n* = 21) receiving an allograft with an HLA-E*01:03/*01:03 genotype (*p* = 0.0237; HR: 3.0, 95% CI: 1.1–7.8, Figure 4B). HLA-E recipient genotypes (Figure 4A) or HLA-E mismatch situation were not relevant for the OS post-HSCT (data not shown).

### sHLA-E Levels Decrease in ATG-Treated Patients, and Subgroup Analysis Confirms sHLA-E Association With Severe aGvHD, Extended cGvHD and Inferior OS Only in Non-ATG Treated Patients

**Figure 5:**
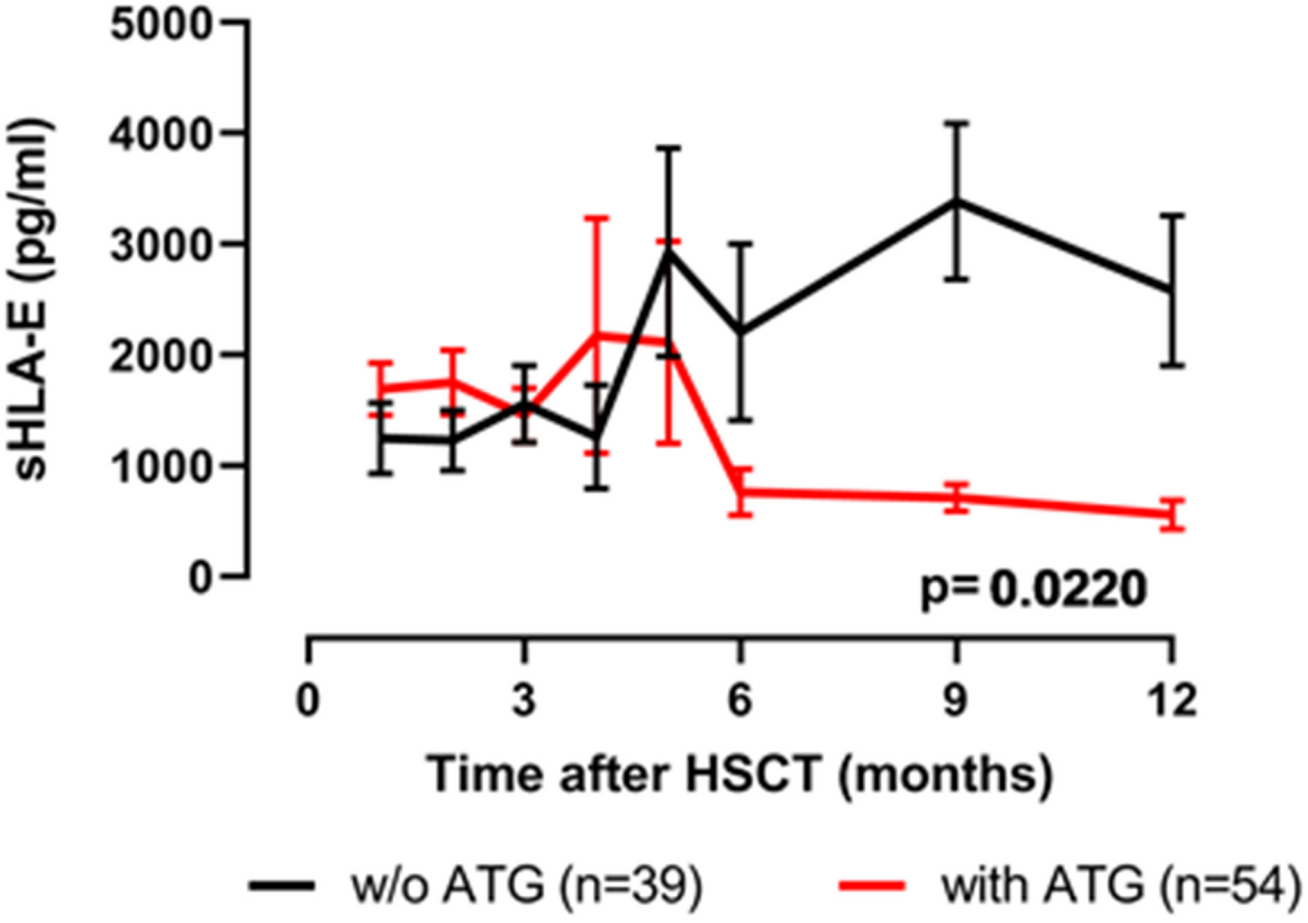
Effects of ATG-conditioning on the course of sHLA-E levels post-HSCT. Patients' sHLA-E levels were divided into two groups according ATG conditioning. Data were presented as mean ± SEM.

**Figure 6:**
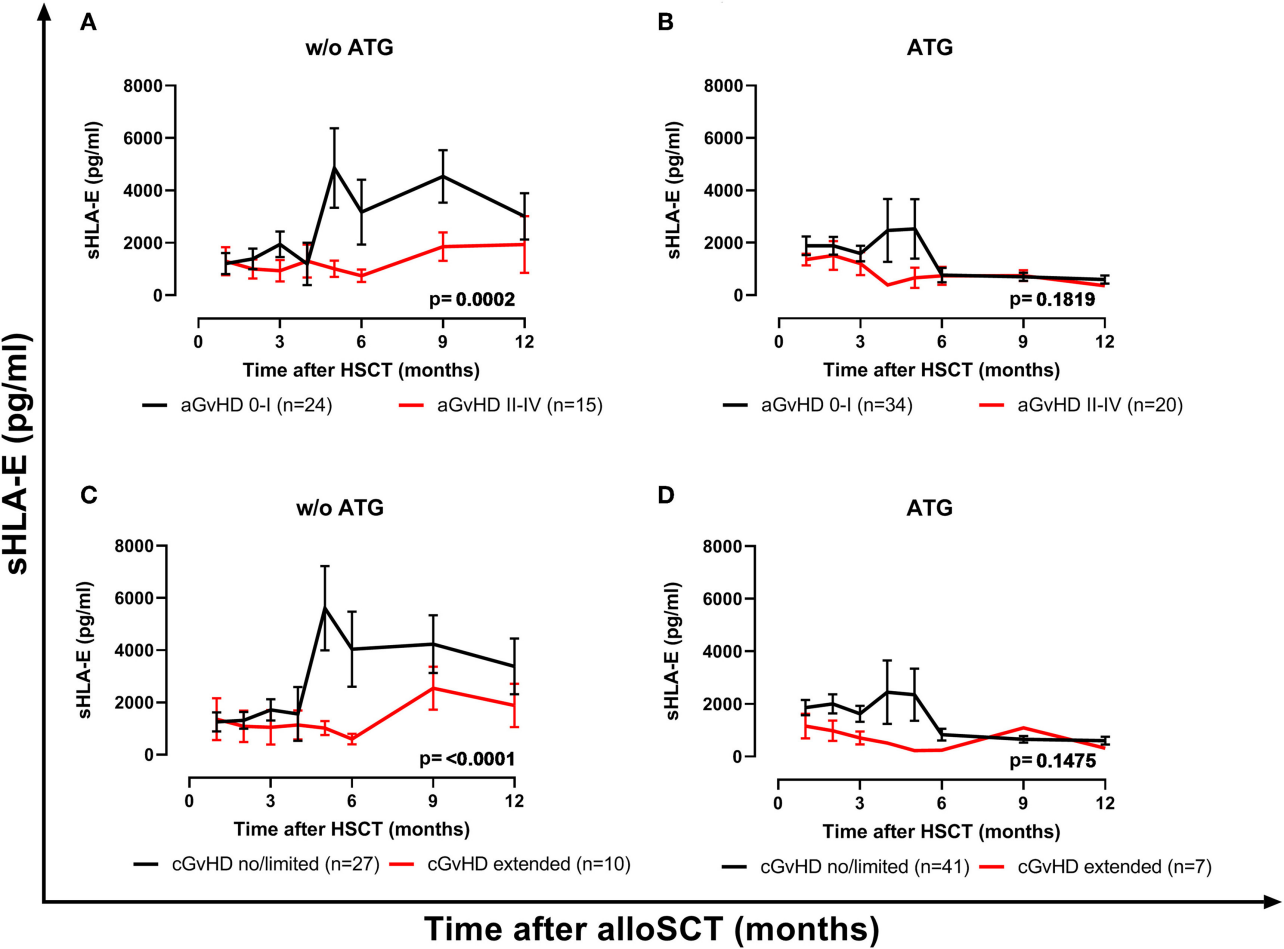
The impact of ATG conditioning on the course of sHLA-E levels with respect to GvHD. sHLA-E levels in non-ATG-treated patients **(A,C)** and ATG-treated **(B,D)** were stratified according to the manifestation of severe aGvHD **(A,B)** or extended cGvHD **(C,D)**. In patients without (w/o) ATG treatment **(A,C)** but not in ATG-treated patients **(B,D)** sHLA-E levels were significantly lower in patients experiencing aGvHD grade II–IV (red line) or extended cGvHD compared to patients with aGvHD patients grade 0–I (black line) or no/limited cGvHD. Manifestation of aGvHD or cGvHD could not be evaluated for all patients due to death or missing clinical data.

**Figure 7:**
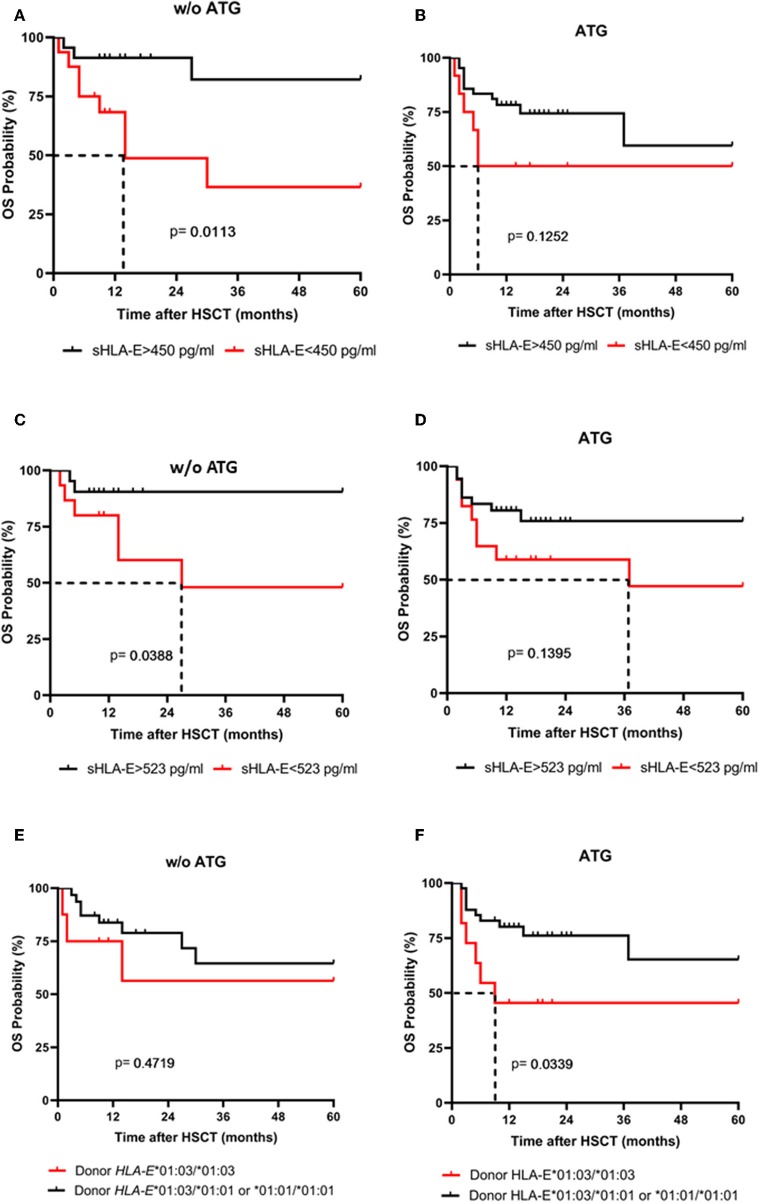
The impact of ATG conditioning on the association sHLA-E levels and donor HLA-E genotype with 5-year OS probability. 5-years OS probabilities were analyzed by Kaplan-Meier analysis combined with log-rank test in patients without (w/o) ATG **(A,C,E)** and with ATG treatment **(B,D,F)** concerning the sHLA-E cut-off levels obtained 1 **(A,B)** and 2 month(s) **(C,D)** post-HSCT and concerning the donor HLA-E genotype status **(E,F)**. Dashed lines indicate the median survival of patients post-HSCT. Only in patients without (w/o) ATG treatment **(A,C)** a significantly lower 5-year probability was evident for patients with sHLA-E levels below the thresholds **(A,C)**. Contrary to sHLA-E threshold levels, a significantly reduced 5-year OS probability was only found for ATG-treated patients receiving a HLA-E*01:03/01:03 allograft.

Next, the influence of ATG treatment on sHLA-E levels after HSCT was analyzed. Comparing ATG-treated patients (*n* = 54) with patients without ATG treatment (*n* = 39) revealed that in the ATG-treated cohort sHLA-E levels started to decrease from month 6 post-HSCT, whereas in non-ATG treated patients the sHLA-E started to increase at that time point (*p* = 0.0220, Figure 5). Although lower sHLA-E levels were observed in ATG-treated and non-ATG-treated patients experiencing acute GvHD grade II-IV or extended chronic GvHD (Figures 6A–D), significant associations of low sHLA-E plasma levels with the clinical endpoints aGvHD (*p* = 0.0002) and cGvHD (*p* < 0.0001) were only observed in the non-ATG-cohort during the time of observation of 12 months. Finally, only in the non-ATG-treated cohort patients with low sHLA-E levels (<450 or <523 pg/mL) showed significantly inferior 5-year OS (*p* = 0.0113 and *p* = 0.0388, respectively, Figures 7A–D). At variance to sHLA-E, the donor HLA-E*01:03/*01:03 genotype was exclusively associated with an impaired 5-year OS (*p* = 0.0339, HR: 4.1, 95% CI: 1.1–15.4) in the ATG-treated cohort, but not in the untreated patient group (Figures 7E,F).

### Low sHLA-E Levels Post-HSCT Were Confirmed as an Independent Marker for Extended cGvHD and OS in Multivariate Analysis

To establish the prognostic relevance of sHLA-E levels post-HSCT or of HLA-E genotype for the prediction of extended cGvHD, binomial logistic regression analysis was performed including ATG treatment as co-variate besides other risk factors such as age at time point of HSCT, ATG treatment, disease status, gender mismatch, HLA-mismatched HSCT, donor, and recipient HLA-E*01:03/01:03 status, and sHLA-E status using the different cut-off levels (450, 523, or 652 pg/mL) obtained from month 1, 2, or 3 post-HSCT as co-variates (Tables 3A–C). Irrespective of the sHLA-E threshold the sHLA-E status was found as an independent predictor for the development of extended cGvHD post-HSCT (*p* = 0.012, HR: 4.5, 95% CI: 1.3–14.7; *p* = 0.021, HR: 3.8, 95% CI: 1.2–12.1; *p* = 0.009, HR: 6.2, 95% CI: 1.5–24.6).

**Table 3:** Multivariate analysis for the prediction of extended cGvHD.

**Risk factors**		**cGvHD**	
		***p***	**HR (95% CI)**
**(A)**			
Age		0.672	1.0 (0.9–1.0)
ATG	Yes	0.617	1.3 (0.4–4.9)
	No		
Female to male HSCT	Yes	0.978	1.0 (0.2–6.9)
	No		
HLA-identical HSCT	Yes	0.111	0.3 (0.1–1.3)
	No		
sHLA-E status	<450 pg/ml	**0.012**	**4.5 (1.3–14.7)**
	>450 pg/ml		
Donor HLA-E^*^01:03/^*^01:03 status	Yes	0.415	1.8 (0.4–8.2)
	No		
Recipient HLA-E^*^01:03/^*^01:03 status	Yes	0.903	1.1 (0.2–8.2)
	No		
Disease status	Early/intermediate	0.048	8.7 (1.0–74.8)
	Late		
**(B)**			
Age		0.755	1.0 (0.9–1.0)
ATG	Yes	0.304	1.8 (0.5–6.3)
	No		
Female to male HSCT	Yes	0.845	1.2 (0.2–7.9)
	No		
HLA-identical HSCT	No	0.546	0.6 (0.1–3.0)
	Yes		
sHLA-E status	<523 pg/ml	**0.021**	**3.8 (1.2–12.1)**
	>523 pg/ml		
Donor HLA-E^*^01:03/^*^01:03 status	Yes	0.143	2.8 (0.7–11.5)
	No		
Recipient HLA-E^*^01:03/^*^01:03 status	Yes	0.957	0.9 (0.1–6.8)
	No		
Disease status	Early/intermediate	0.100	5.9 (0.7–50.1)
	Late		
**(C)**			
Age		0.677	1.0 (0.9–1.0)
ATG	Yes	0.131	2.7 (0.7–10.3)
	No		
Female to male HSCT	Yes	0.700	1.4 (0.2–10.7)
	No		
HLA-identical HSCT	No	0.520	0.6 (0.1–3.1)
	Yes		
sHLA-E status	<652 pg/ml	**0.009**	**6.2 (1.5–24.6)**
	>652 pg/ml		
Donor HLA-E^*^01:03/^*^01:03 status	Yes	0.134	3.2 (0.7–14.6)
	No		
Recipient HLA-E^*^01:03/^*^01:03 status	Yes	0.493	1.9 (0.3–14.1)
	No		
Disease status	Early/intermediate	0.192	4.3 (0.5–39.9)
	Late		

*sHLA-E thresholds levels obtained 1 month (A), 2 months (B), or 3 months (C) post-HSCT were used as risk factors. P-values were evaluated by binomial logistic regression analysis*.

*The bold values indicate the significant p-value of the sHLA-E threshold*.

Moreover, multivariate analysis using Cox regression including the same co-variates revealed that the sHLA-E status with a threshold value of 523 pg/mL (*p* = 0.041, HR: 2.1, 95% CI: 1.0–4.7) was an independent predictive marker for OS, whereas the sHLA-E cut-off of 450 pg/mL (*p* = 0.054, HR: 2.1, 95% CI: 1.0–4.7) did not reach significance (Tables 4A,B). The HLA-E genotypes of donor or recipient were not found to be of prognostic relevance for both, the prediction of cGvHD or OS post-HSCT (Tables 4A,B).

**Table 4:** Multivariate analysis for the prediction of 5-year OS.

**Risk factors**		**5-Year OS**	
		***p***	**HR (95% CI)**
**(A)**			
Age		0.262	1.0 (0.9–1.0)
ATG	Yes	0.465	0.7 (0.3–1.7)
	No		
Female to male HSCT	Yes	0.047	2.5 (1.0–6.2)
	No		
HLA-identical HSCT	Yes	0.057	2.3 (0.9–5.8)
	No		
sHLA-E status	<450 pg/ml	**0.054**	**2.1 (1.0–4.7)**
	>450 pg/ml		
Donor HLA-E^*^01:03/^*^01:03 status	Yes	0.087	2.2 (0.9–5.5)
	No		
Recipient HLA-E^*^01:03/^*^01:03 status	Yes	0.619	1.4 (0.4–5.3)
	No		
Disease status	Early/intermediate	0.048	0.4 (0.2–1.0)
	Late		
**(B)**			
Age		0.183	1.0 (1.−1.1)
ATG	Yes	0.793	0.9 (0.3–2.7)
	No		
Female to male HSCT	Yes	0.510	1.5 (0.5–4.5)
	No		
HLA-identical HSCT	No	0.117	1.8 (0.8–4.3)
	Yes		
sHLA-E status	<523 pg/ml	**0.041**	**2.1 (1.0–4.7)**
	>523 pg/ml		
Donor HLA-E^*^01:03/^*^01:03 status	Yes	0.156	1.9 (0.8–4.3)
	No		
Recipient HLA-E^*^01:03/^*^01:03 status	Yes	0.653	1.3 (0.3–5.2)
	No		
Disease status	Early/intermediate	0.041	0.4 (0.2–1.0)
	Late		

*sHLA-E thresholds levels obtained at 1 month (A) and 2 months (B) were used as risk factor. P-values were evaluated by Cox Regression analysis*.

*The bold values indicate the significant p-value of the sHLA-E threshold*.

## Discussion

In recent years, the non-classical HLA-molecules have gained increasing attention with respect to their role regarding clinical endpoints in HSCT. We have previously investigated the influence of the non-classical HLA-molecule HLA-G after HSCT and observed in 32 patients a correlation of elevated soluble HLA-G levels with less severe acute or chronic GvHD, and with a superior overall survival (23). Here, we present data on the role of the non-classical HLA-molecule HLA-E in a larger cohort of 93 patients. In summary, (a) we observed that severe acute GvHD grade II-IV, extended chronic GvHD, and inferior OS are associated with reduced sHLA-E plasma levels during the first year post-HSCT; (b) we identified specific sHLA-E cut-off levels obtained 1, 2, or 3 month(s) post-HSCT related to severe aGvHD grade II-IV and extended cGvHD as well as to inferior OS by ROC analysis; (c) we found no association of sHLA-E levels post-HSCT with the recipient or with the donor HLA-E genotype; (d) we evidenced an inferior 5-year OS for patients receiving an allograft with HLA E*01:03 homozygosity; (e) in subgroup analyses, the association of severe acute and extended chronic GvHD and of inferior OS with diminished sHLA-E plasma levels could be confirmed only in non-ATG-treated patients, but not in patients treated with ATG; (f) multivariate analyses including ATG treatment as co-variate among other risk factors confirmed low sHLA-E levels but not donor HLA E*01:03 homozygosity as an independent predictor for extended cGvHD and OS.

Studies investigating the role of HLA-E in the context of HSCT have produced inconsistent results regarding the influence of HLA-E genotypes on clinical endpoints like TRM, acute and chronic GvHD, relapse, disease free, and overall survival after HSCT (12–17). Tamouza et al. described already in 2006 in 187 matched related HSCT a lower incidence of aGvHD and TRM at day 180 when the genotype was HLA-E*01:03/E*01:03, either in the donor or in the recipient (17). Danzer et al. confirmed the association of HLA*103 homozygosity with a significantly decreased incidence of TRM and in addition also an improved DFS and OS in a cohort of 83 related and unrelated HSCT (12). The authors attribute these beneficial effects to the prevention of NK-cell dependent lysis by HLA-E interaction with CD94/NKG2A. Finally, Hosseini et al. observed a lower frequency of acute GvHD (grade II or more; *p* = 0.02) and extensive chronic GvHD (*p* = 0.04) (14), besides also a lower TRM and better OS in 56 patients with HLA-E*01:03/01:03 genotype (16).

These findings, however, were challenged by an analysis of 116 HSCT patients and their matched unrelated donors published in 2012 (13). Fürst et al. report that neither univariate nor multivariate analysis shows any influence of HLA-E polymorphisms on acute GvHD, TRM, DFS, or OS. In contrast to the early publications, Tsamadou et al. in 2017, too, could not confirm the association of HLA-E 01:03/01:03 with better outcome; they rather described a worse outcome albeit without reaching statistical significance (21). Moreover, according to this study HLA-E mismatch was significantly associated with improved NRM, DFS, and OS, especially patients with advanced disease stage benefit from HLA-E mismatch. However, the same authors could not confirm the putative beneficial effect of HLA-E mismatch between donor and recipient in a larger cohort analyzed 2 years later (22). In this study, the authors observed a significant association of donor and recipient HLA-E*01:03/01:03 homozygosity with worse DFS and higher risk of relapse. Specifically, according to the authors, the donor genotype is mainly driving the effect, however, only in a T cell replete setting; *in vivo* T cell depletion (with ATG or campath) abrogates the effect of donor HLA-E genotype.

In contrast to the aforementioned publications, which obviously have produced contradictory results and conclusions, we assume that rather the factual measurable amount of soluble HLA-E exerts more influence on immune modulation. Hence, the clinical endpoints of HSCT like acute and chronic GvHD and OS have to be associated rather with sHLA-E than with HLA-E polymorphisms. To test this hypothesis, we determined the soluble HLA-E levels and enquired any association with these and the clinical endpoints, which produced the results we have reported. In our study, the pre-HSCT levels appeared to be independent from gender, HLA-E genotype, and type of disease of patients. So far, increased sHLA-E levels have been described for chronic lymphocytic leukemia (11) and acute leukemia patients (31) compared to healthy individuals. Nevertheless, the mean pre-HSCT level of our patients (1,352 ± 210 SEM pg/ml) were in range of the reported mean sHLA-E levels (1,222 ± 101) of healthy individuals (11). Most importantly, we could not detect any association of the course of sHLA-E levels post-HSCT with the three HLA-E genotypes (HLA-E*01:03/*01:03, HLA-E*01:01/*01:01, HLA-E*01:01/*01:03). Consequently, the course of sHLA-levels can be enquired independently of the HLA-E genotype. Notably, we observed significant associations of sHLA-E plasma levels with clinical endpoints after HSCT. Reduced sHLA-E plasma levels appear to increase the risk for severe acute GvHD and even more pronounced for chronic GvHD and overall survival in all univariate analyses. However, this effect is obviously derogated when ATG conditioning of the patients is taken into account: a strong association of low sHLA-E levels with aGvHD, cGvHD, and inferior 5-years OS was observed only in patients without ATG-treatment, whereas the donor HLA-E*01:03 homozygosity was only related with reduced OS in patients treated with ATG. The latter finding is at variance to a recent study of Tsamadou and colleagues, who report an abrogation of the effect of donor HLA-E genotype in ATG-treated patients (22), although we confirm the observations of this study group regarding survival, as we describe an association of donor HLA-E*01:03 homozygosity with inferior 5-year-OS (21, 22). Regarding relapse, we did not observe any association of the course of sHLA-E levels post-HSCT with the occurrence of relapse following HSCT.

The findings reported here emerged out of prospectively collected data of patient population with as well-unrelated and related HSCT and with a variety of hematological diseases. We paid attention to both HLA-E genotypes and amounts of soluble HLA-E molecules. However, limitations of our study can result from a single-center effect, limited number of patients, and limited median follow-up time of roughly 14 months. Yet, taken together our findings shed some light on the impact of the non-classical HLA molecule HLA-E in HSCT and specifically associates decreased levels of soluble HLA-E with a higher incidence of severe acute and chronic GvHD and inferior OS. Our data could be useful to develop effective strategies for the prediction and prevention of HSCT complications and besides, be instrumental to identify patients who might be candidates for a reduction in immunosuppressive treatment, which is particularly important in patients with high relapse risk. Obviously, these data have to be validated in larger patient cohorts and its pathophysiological functionality ought to be further elucidated in mechanistical *in vivo* and *in vitro* models.

## Data Availability Statement

The datasets generated for this study are available on request to the corresponding author.

## Ethics Statement

The studies involving human participants were reviewed and approved by Ethical Board of the University Hospital of Essen, Germany (07-3503). The patients/participants provided their written informed consent to participate in this study.

## Author Contributions

VR and LK conceived, designed, and performed the experiments, analyzed the data and wrote the manuscript. ES, ML, FH, UB, PH, and DB confirmed the analyses and assisted in correcting the manuscript.

### Conflict of Interest

The authors declare that the research was conducted in the absence of any commercial or financial relationships that could be construed as a potential conflict of interest.
