# Supplementary material for: Decreased Soluble Human Leukocyte Antigen E Levels in Patients After Allogeneic Hematopoietic Stem Cell Transplantation Are Associated With Severe Acute and Extended Chronic Graft-versus-Host Disease and Inferior Overall Survival
Source: Front Immunol. 2020 Jan 10;10:3027. doi: 10.3389/fimmu.2019.03027 (PMC6966962; doi:10.3389/fimmu.2019.03027)
Supplement: Supplementary file 1 [file Data_Sheet_1.pdf]

Supplementary Figures

Supplementary Figure 1: No association of Pre-HSCT sHLA-E levels with gender, HLA-E genotypes, type of disease, and sHLA-E levels at one month post-HSCT. sHLA-E levels are shown by Box-Whisker plots with 10 and 90 percentiles.

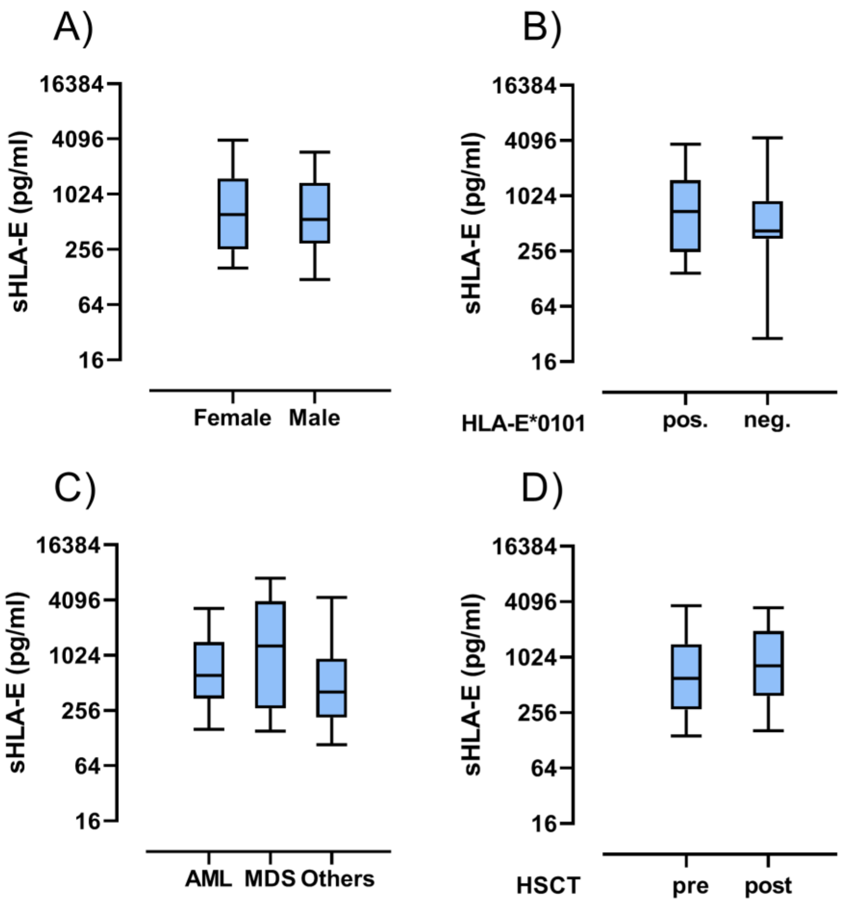

Supplementary Figure 2: Course of sHLA-E levels post HSCT during the 12 months.

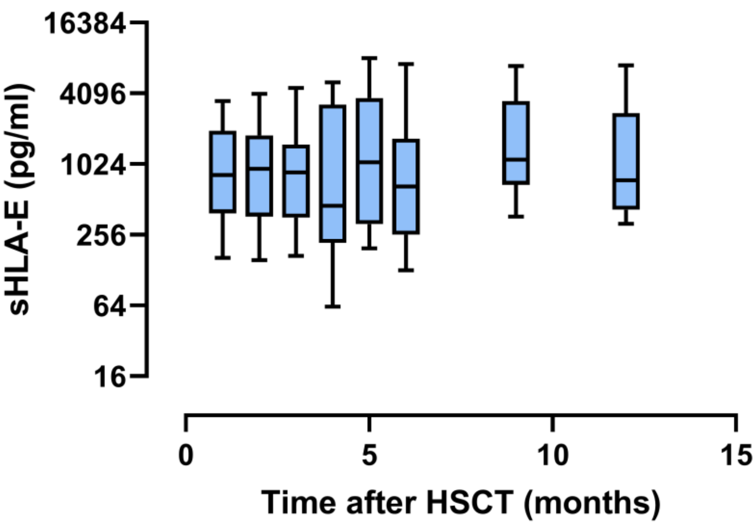

Supplementary Figure 3: Receiver operating characteristic (ROC) analyses for the threshold determination of sHLA-E levels at various blood sampling time points post HSCT regarding aGvHD, cGvHD or OS. Black, green, or red lines indicates ROC for sHLA-E levels obtained one, two, and three month(s), respectively.

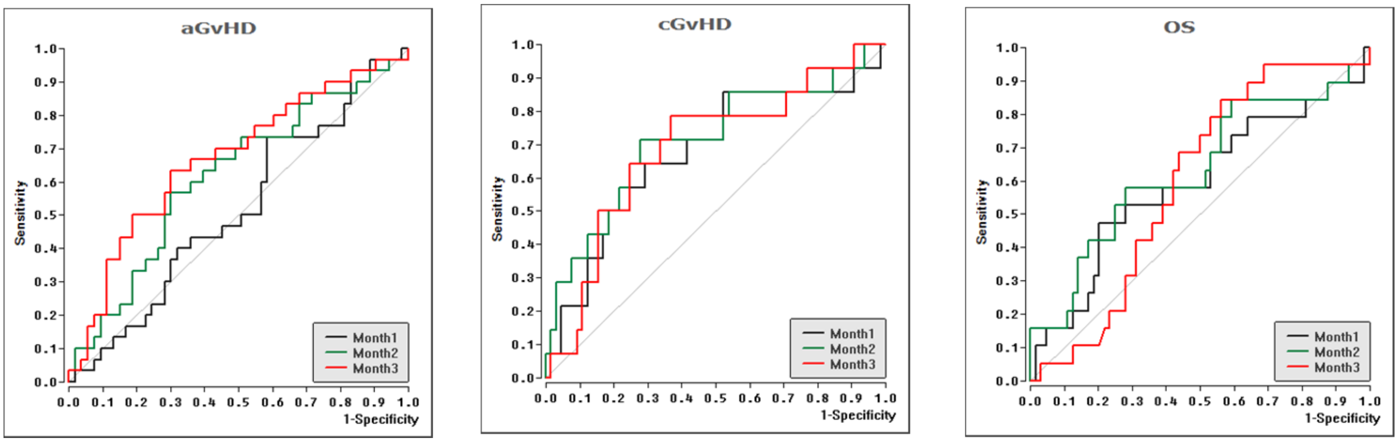

Supplementary Tables

Supplementary Table 1: sHLA-E cut-off levels defined by ROC analysis for aGvHD, cGvHD and 5-year OS. P-values were defined by Mann-Whitney test.

HSCT endpoint	Month	Cut-off	AUC	Sensitivity	Specificity	p
**aGvHD**	1	1595	0.502	74.3	37.9	0.968
	2	608	0.611	55.9	69.1	0.084
	3	652	0.655	63.3	68.5	0.019
**cGvHD**	1	450	0.627	52.9	76.4	0.097
	2	523	0.660	62.5	71.6	0.047
	3	652	0.660	78.5	62.1	0.037
**OS**	1	450	0.639	50.0	78.5	0.032
	2	523	0.651	58.3	72.3	0.028
	3	1244	0.611	85.0	43.8	0.134

Supplementary Table 2: Association of HLA-E genotypes with the 5-year overall survival

Recipient HLA-E genotype	OS alive	OS death	p	OR	95% CI
**01:01/01:01**	18 (27.5%)	10 (35.7%)	0.467	1.45	0.57-1.75
**01:01/01:03**	35 (53.8%)	9 (32.1%)	0.071	2.46	0.95-5.87
**01:03/01:03**	12 (18.5%)	9 (32.1%)	0.179	2.09	0.77-5.44
**Allele frequencies**					
**01:01**	71	29	0.752	1.12	0.60-2.08
**01:03**	59	27			
**Donor HLA-E genotype**	**OS alive**	**OS death**	**p**	**OR**	**95% CI**
**01:01/01:01**	21 (32.8%)	8 (29.6%)	0.810	1.16	0.45-3.14
**01:01/01:03**	33 (51.6%)	10 (37.0%)	0.178	2.02	0.80-5.27
**01:03/01:03**	10 (15.6%)	9 (33.3%)	0.088	2.70	0.95-7.48
**Allele frequencies**					
**01:01**	75	26	0.253	1.52	0.80-2.92
**01:03**	53	28			
